# The morphology of the preimaginal stages of *Squamapion elongatum* (Germar, 1817) (Coleoptera, Curculionoidea, Apionidae) and notes on its biology

**DOI:** 10.3897/zookeys.519.9134

**Published:** 2015-09-01

**Authors:** Jacek Łętowski, Krzysztof Pawlęga, Radosław Ścibior, Karol Rojek

**Affiliations:** 1 Department of Zoology, Animal Ecology and Wildlife Management,University of Life Sciences in Lublin, Lublin, Poland; 2 Department of Applied Pharmacy, Medical University of Lublin, Lublin, Poland

**Keywords:** Weevil, Apionidae, *Squamapion*, egg, mature larva, pupa, life cycle, *Salvia*, Lamiaceae

## Abstract

Data on the morphology of the egg, mature larva (L_3_) and pupa of *Squamapion elongatum* (Germar, 1817) are presented. The development cycle of this species lasts 51–54 days: a 12-day egg period, a 30-day larval period, and a 12-day pupal period, on average. The larvae are attacked by parasitic hymenopterans of the superfamily Chalcidoidea.

## Introduction

The genus *Squamapion* Bokor, 1923 is distributed in the Palaearctic and Afrotropical regions and includes 33 species (Alonso-Zarazaga 2011). In Poland it is represented by 9 species: *Squamapion atomarium* (Kirby, 1808), *Squamapion cineraceum* (Wencker, 1864), *Squamapion elongatum* (Germar, 1817), *Squamapion flavimanum* (Gyllenhal, 1833), *Squamapion serpyllicola* (Wencker, 1864), *Squamapion mroczkowskii* (Wanat, 1997), *Squamapion oblivium* (Schilsky, 1902), *Squamapion samarense* (Faust, 1891) and *Squamapion vicinum* (Kirby, 1808) (Petryszak 2004; Mokrzycki and Wanat 2005). These are mono- or oligophagous herbivores feeding on species from the family Lamiaceae, mainly of the genera *Salvia*, *Thymus*, *Thymbra*, *Mentha*, *Origanum*, *Prunella* and *Saccocalyx*. Their larvae burrow tunnels inside roots or stems, occasionally causing galls (Alonso-Zarazaga 1990). These insects are quite similar in terms of external morphology, which causes many problems in identification. As yet no attempts have been made to divide the taxon into subgenera or species groups (Wanat 1997).

*Squamapion elongatum* is a species inhabiting the southern and central part of Europe, as well as East Asia and Algeria. It inhabits lowland and submontane areas. In Poland it is recorded in the Masurian Lake District, the Wielkopolsko-Kujawska Lowland, Upper and Lower Silesia, the Krakowsko-Wieluńska Upland, the Małopolska Upland, the Świetokrzyskie Mountains, the Lublin Upland, Roztocze and Eastern Beskid (Burakowski et al. 1992). This species is characteristic of xerothermic grasslands, where it feeds on plants of the genus *Salvia* – *Salvia pratensis* and *Salvia nemorosa* (Cmoluch 1962). Its life cycle has not yet been described.

## Material and methods

*Squamapion elongatum* eggs, larvae, pupae and adults were collected from two patches of xerothermic grasslands in Gródek (50°46'58.18"N, 23°56'47.04"E) near Hrubieszów and in Łęczna (51°18'9.7"N, 22°51'47.8"E) (SE Poland). The material was collected during one growing season from July to September 2011 at 3–7 day intervals between 10 a.m. and 2 p.m. Adults were collected using an entomological net in an association of *Thalictro*-*Salvietum pratensis*. To obtain the other development stages for breeding, whole plants of the genus *Salvia* were collected. In total 67 specimens of meadow sage were collected. A delicate cut was made along the stem and root of the plants and then they were dissected with needles to find the eggs, larvae, pupae and even adults located inside. Some of the larvae were used to begin breeding and others for microscopic slides, to be used in making drawings showing the morphology of the developmental stages. To prepare the drawings we used an OLYMPUS BX61 microscope at magnifications from 200 × to 400 × and a TESCAN VEGA3LMU scanning microscope at magnifications from 500 × to 4,500 ×. The figures were made based on the biological preparations using Corel Draw 12 software. Metric sizes are the average value of 10 measurements (Table 1).

**Table 1.** The measurement values of length and width of larvae (L_3_) and pupae bodies.

No	Larva (L_3_)		Pupa	
	length	width	length	width
1	2.80	1.28	2.69	0.93
2	2.76	1.24	2.68	0.95
3	2.81	1.22	2.67	0.96
4	2.75	1.23	2.66	0.92
5	2.78	1.26	2.65	0.93
6	2.77	1.25	2.69	0.98
7	2.79	1.24	2.65	0.95
8	2.81	1.22	2.67	0.94
9	2.75	1.26	2.69	0.93
10	2.78	1.24	2.67	0.95
average	2.78	1.24	2.67	0.94

Larval specimens in successive developmental stages were transferred in vitro to Petri dishes after the larval stadium was determined on the basis of morphological characteristics and the number of exuvia of head capsules. Breeding of larvae was carried out to the L_3 _stage. Breeding of preimaginal stages was carried out according to Scherf (1964) and Łętowski (1991). Petri dishes were transferred to a breeding chamber with the following conditions: temperature during the day 30 °C, temperature at night 20 °C, humidity during the day 60%, humidity at night 80%. Adults were kept in glass containers covered with a fine mesh. As in the case of the larvae, filter paper saturated with distilled water was placed at the bottom to maintain humidity and as a possible reservoir of drinking water for the beetles. They were fed with fragments of fresh sage shoots, which were replaced on average every three days. The used stems were examined to search for eggs. Microscope slides with the developmental stages and their morphological structures were prepared according to Łętowski (1991) and Gosik et al. (2010). The terminology of Scherf (1964), May (1993, 1994), Marvaldi (2003) and Wang et al. (2013) was used in the morphological description of the larva and pupa. The morphology of the egg, L_3_ and pupa and the developmental cycle from egg to adult were described.

Setae of thorax and abdomen of larva (L_3_) and pupa are described for one side only.

## Description

**Egg** (Figure 11)

Length ca. 1.13 mm, width ca. 0.57 mm, oval, smooth, shiny, whitish-yellow.

**Figure 1.**
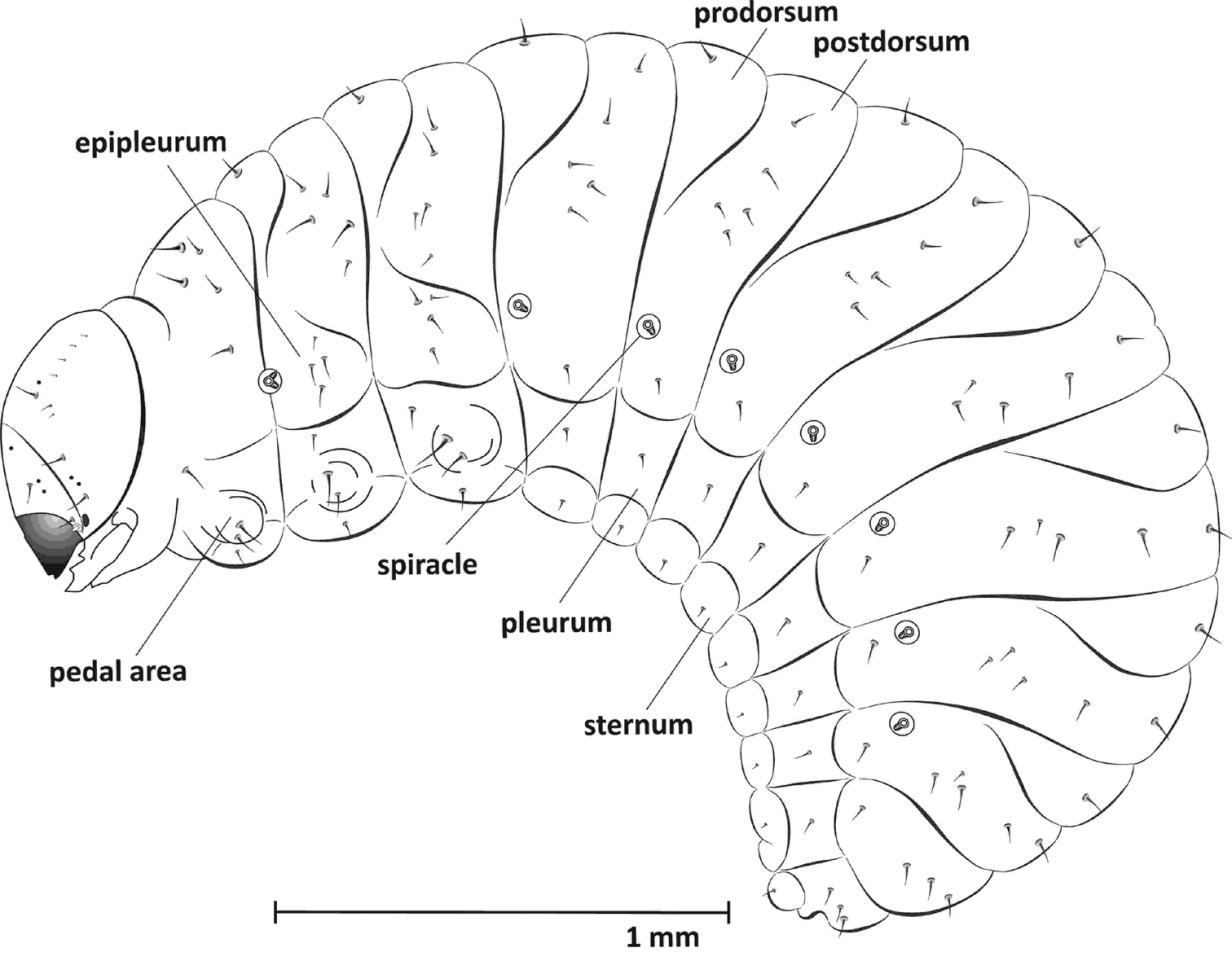
Mature larva (L_3_), lateral view.

**Mature larva (L_3_)** (Figures 1, 12)

Length ca. 2.78 mm, width ca. 1.24 mm. Body massive and strongly curved, whitish-yellow, with short setae.

**Figure 2.**
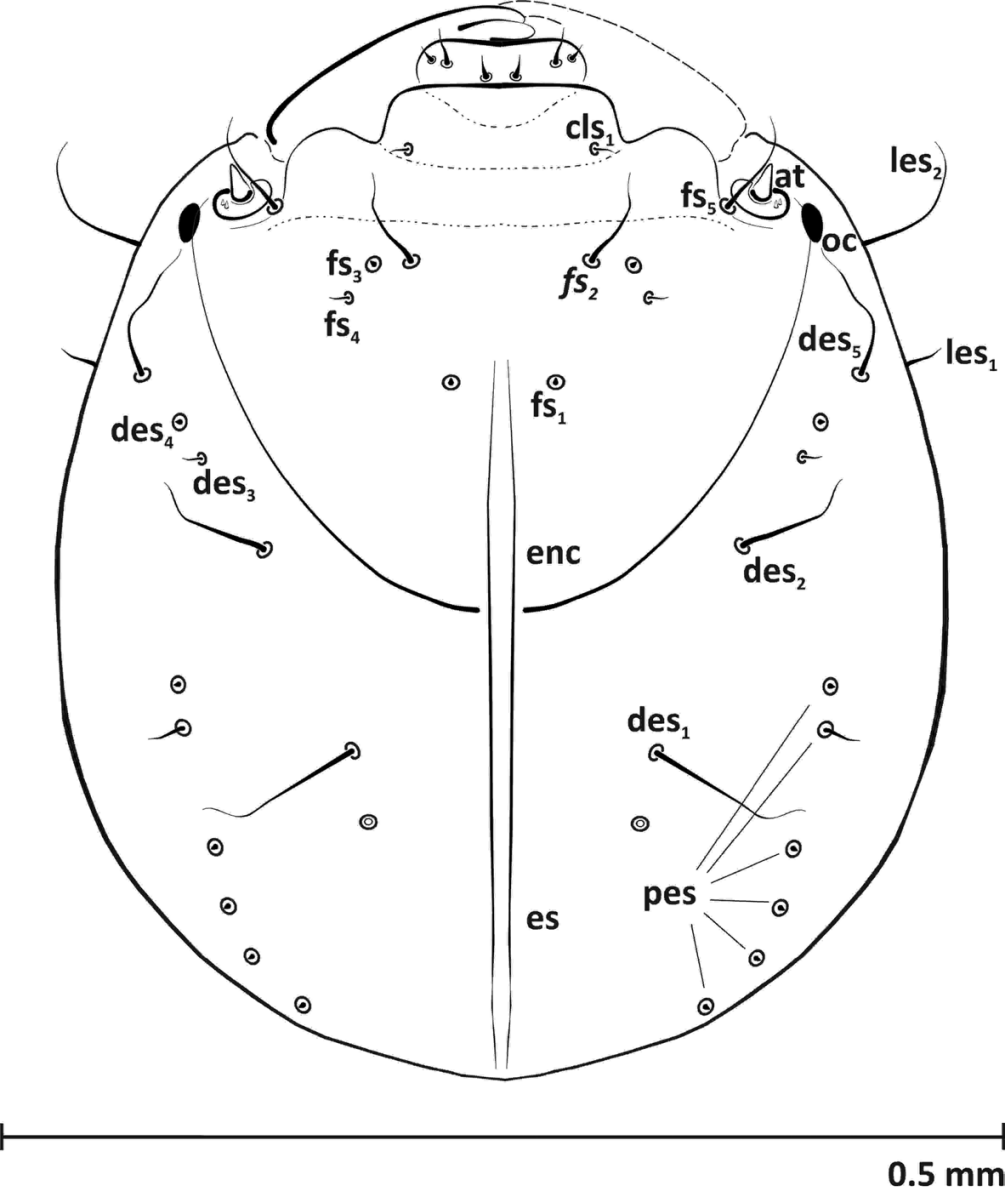
Epicranium (L_3_), dorsal view: ***les*** lateral epicranial setae ***fs*** frontal s. ***des*** dorsal s. ***pes*** posterior epicranial s. ***cls*** clypeus s. ***at*** antenna ***oc*** ocellus ***enc*** endocarina, ***es*** endocarina suture.

**Head** (Figures 2, 9, 10): Yellow-brown, oval, with clear frontal suture completely extended to mandibular joint. Epicranium: length ca. 0.72 mm, width ca. 0.56 mm. Endocarina (*enc*) long, extended to 2/3 the length of the frons. One pair of ocelli (*oc*). Antennae (*at*) with conical sensorium and 2 small spinose sensilla (Fig. 10). Clypeus with 1 pair of short setae (*cls1*). Frons with 5 pairs of setae (*fs*) – *fs2* and *fs5* longer, *fs1*, *fs3* and *fs4* much shorter, at the apex of the endocarina, *fs2*-*4* near the epistoma, *fs5* close to the antennae (Fig. 2). Epicranium with 2 pairs of lateral setae (*les*) – *les1* short, *les2* long, more than 3 times longer than *les1*.; 5 pairs of dorsal setae (*des*) – *des1* very long, *des2* and *des5* a bit shorter and *des3* and *des4* much shorter; 4 pairs of minute posterior setae (*pes*) (in line with *des2*); 1 sensillum between epicranial suture and *pes* and another sensillum laterally to *des1* and close to *des2*.

**Figure 3.**
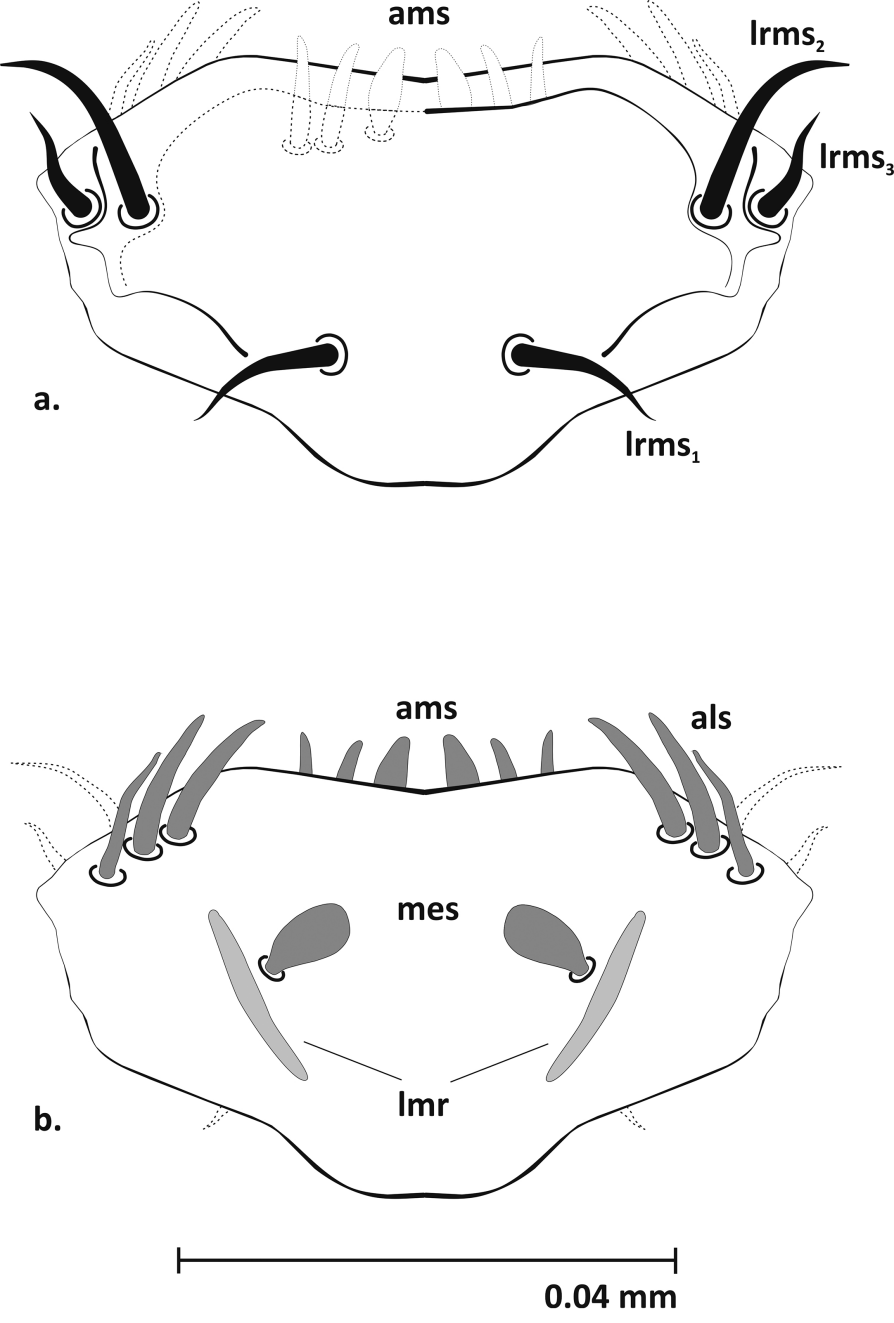
Labrum and epipharynx (L_3_) (**a** dorsal **b** ventral view): **a *lrms*** labral setae **b *mes*** median s. ***ams*** anteromedial s. ***als*** anterolateral s. ***lmr*** labral rods.

**Figure 4.**
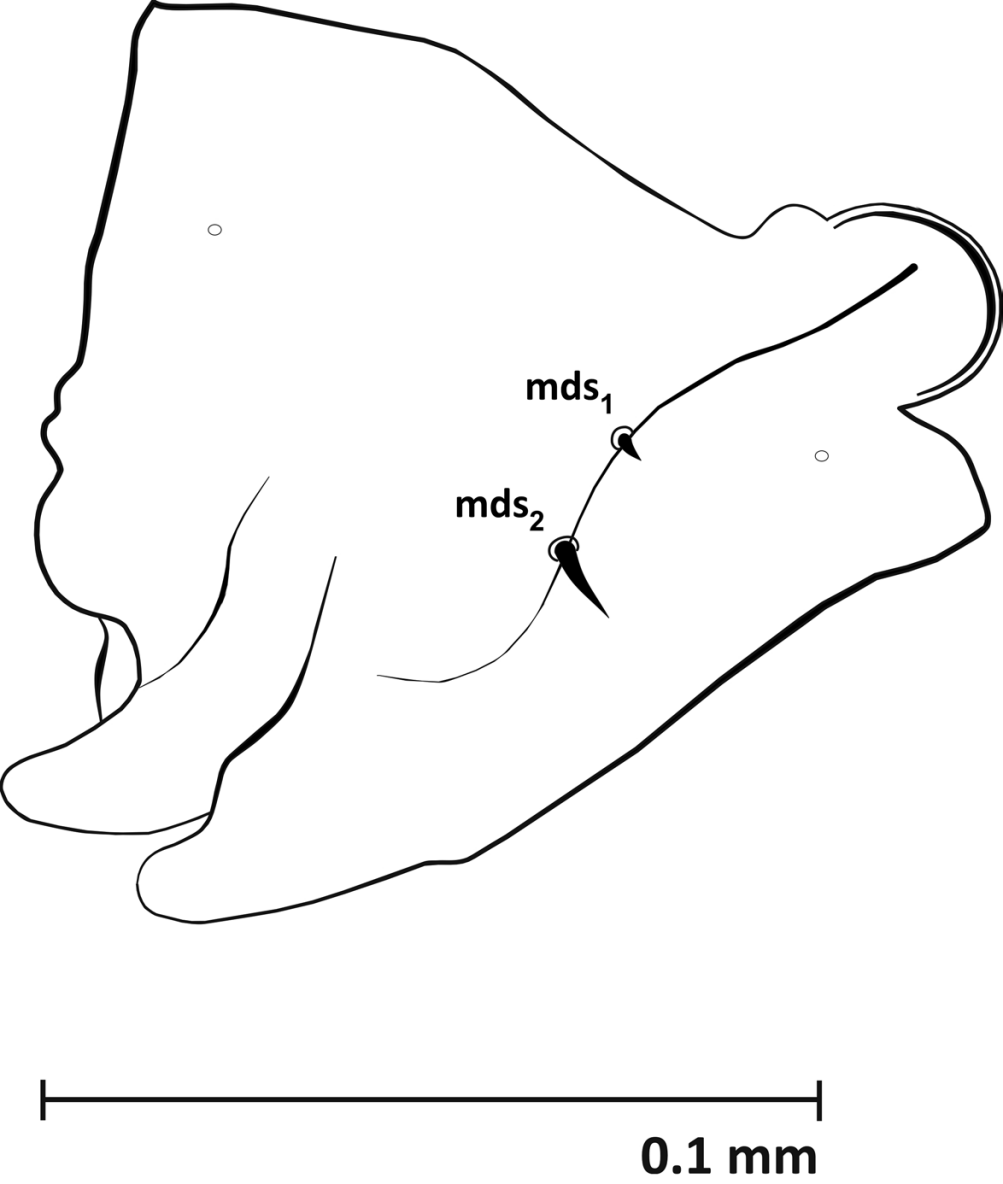
Mandible (L_3_), left: ***mds*** dorsal malae setae.

**Figure 5.**
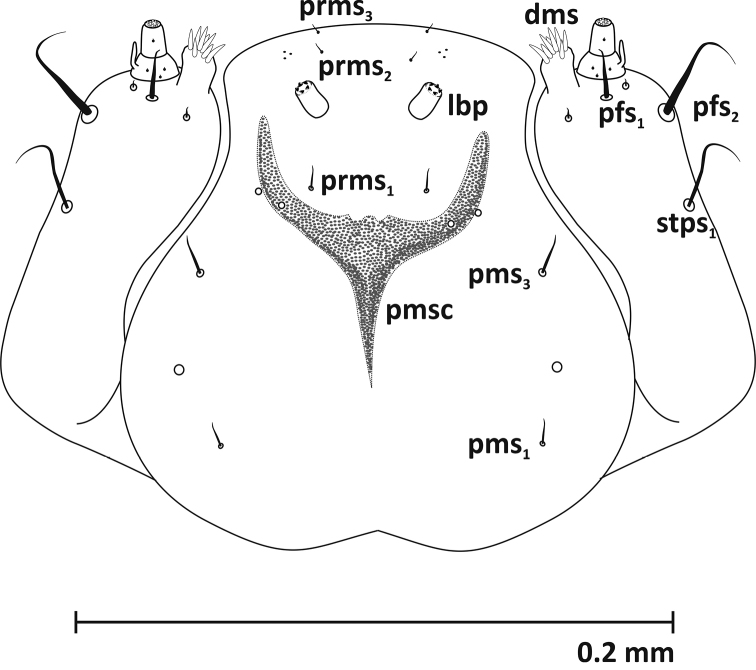
Maxillae and labium (L_3_): ***dms*** dorsal maxillary setae ***pfs*** palpiferal s. ***stps*** stipal s. ***prms*** prelabium s. ***pms*** postlabium s. ***pmsc*** premental sclerite ***lbp*** labial palpus.

**Mouthparts** (Figures 3–5, 9): Dorsal side of labrum with 3 pairs of setae (*lrms1-3*) – *lrms1* and *2* long, *lrms3* nearly half the length of *lrms2*. Epipharynx with 7 pairs of setae (3 *ams*, 3 *als*, 1 *mes*) and rather short labral rods (*lmr*) (Fig. 3a, b). Mandibles massive, dark brown, with 2 mandibular setae (*mds1, 2*) and 2 sensilla (Fig. 4). Maxillae: palpifer with 2 long setae (*pfs1*, *2*) – *pfs2* longer than *pfs1*, 1 longer stipital seta (*stps1*) and 2 very short sensilla. Maxillary palpus with 2 segments. Basal segment with digitiform sensorium and 3 very short setae, distal segment cylindrical with 1 short sensilla and 10 conical papillae. Lacinia with 8 robust setae arranged like a comb (*dms*) (Fig. 5, 9). Labium: premental sclerite (*pmsc*) Y-shaped. Mentum-submentum complex with 2 pairs of postmental setae (*pms1, 3*) and 3 pairs of prelabial setae (*prms1-3*) – *prms1* short, *prms2* and *3* very short. Additionally, submentum with 3 pairs of sensilla. Labial palpi (*lbp*) 1-segmented, raised, with numerous papillae (Fig. 5).

**Thorax.** Pronotal shield unsclerotized, meso- and metanotum each with 2 folds: pro- and postdorsum. Thoracic spiracle intersegmental, in membrane between pro- and mesothorax, bicameral. Prothorax with 7 setae: pronotum with 5, epipleurum indistinct, with 1 seta, sternum with 1 seta. Meso- and metathorax with 12 setae: prodorsum with 1 seta, postdorsum with 5 setae, epipleurum (clearly visible) with 4 clear setae, pleurum and sternum with 1 seta. Pedal area with two long setae for all segments (Fig. 1).

**Abdomen.** Tergites I-VII with 2 folds, prodorsum with 1 seta on the ridge, postdorsum with 6 setae – 5 dorsally located and 1 seta surrounded by a circle of sparse tubercles. Tergites VIII-IX without folds (single), VIII with 4 setae and XI with 3 setae, reduced. Segments I-VII with unicameral spiracles, others without spiracles. Pleura and sterna I-VIII with 1 short seta, sterna IX with 1 short seta (Fig. 1).

**Figure 6.**
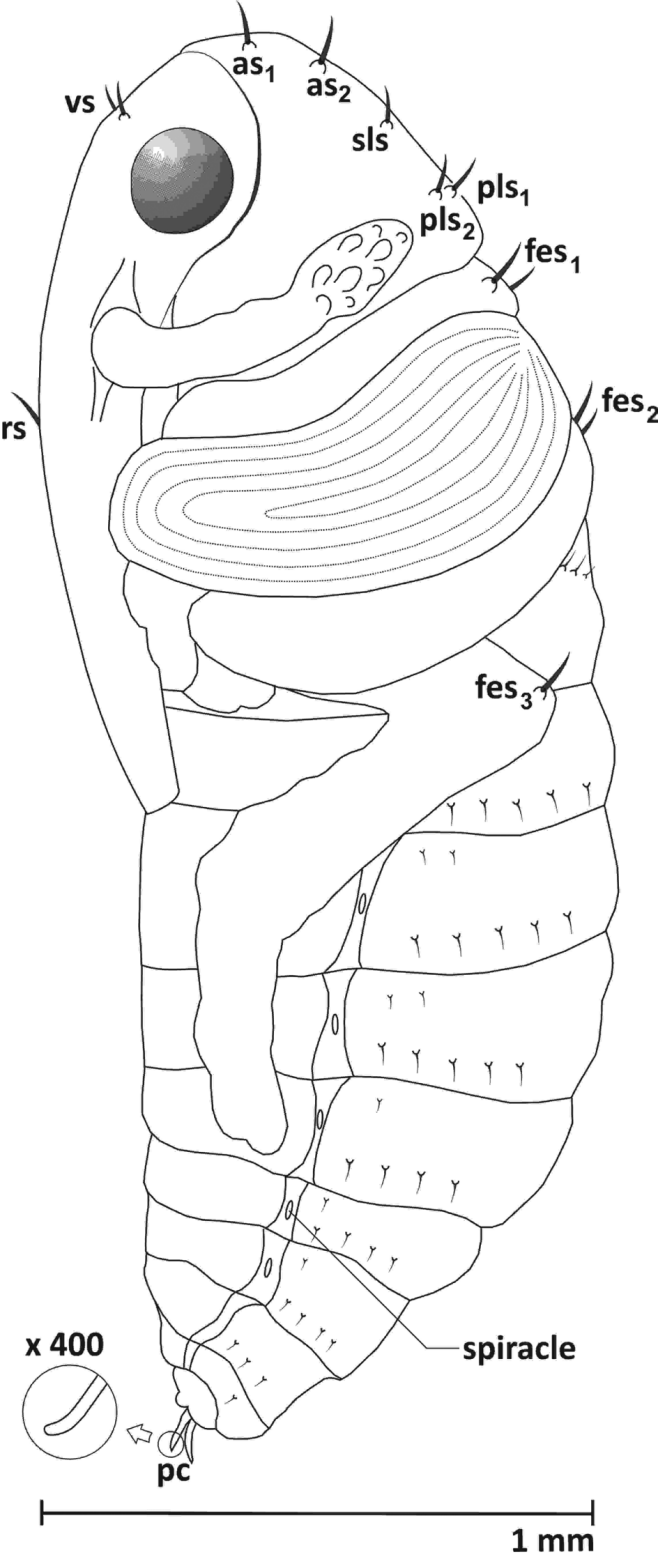
Pupa, lateral view: ***as*** apical s., ***sls*** sublateral s., ***pls*** posterolateral s. ***vs*** vertical s. ***rs*** rostral s. ***fes*** femoral s. ***pc*** urogomphi.

**Figure 7.**
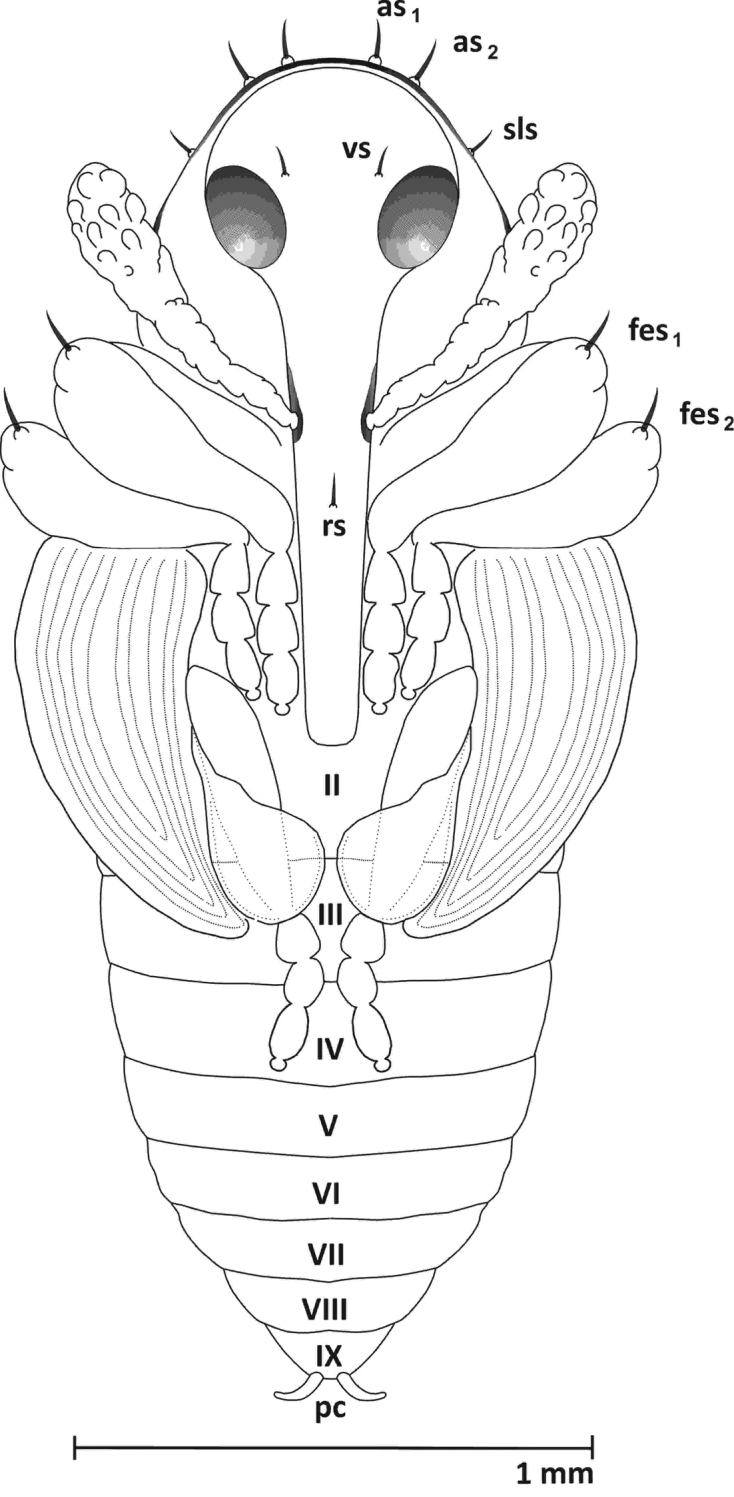
Pupa, ventral view: ***as*** apical s. ***sls*** sublateral s. ***vs*** vertical setae ***rs*** rostral s. ***fes*** femoral s. ***pc*** urogomphi.

**Figure 8.**
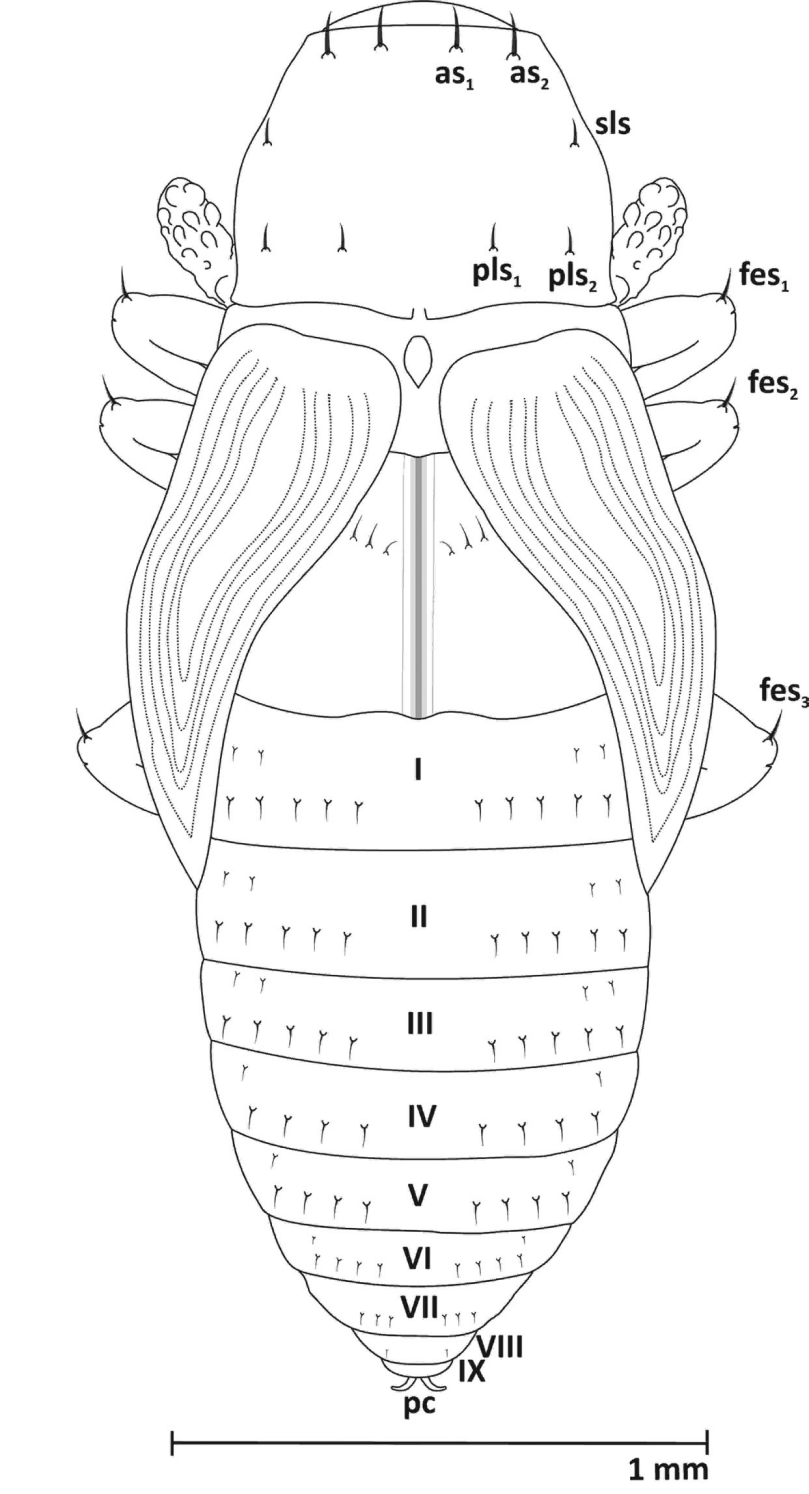
Pupa, dorsal view: ***as*** apical s. ***sls*** sublateral s. ***pls*** posterolateral s. ***fes*** femoral s. ***pc*** urogomphi.

**Pupa** (Figures 6–8, 13)

**Figures 9–14.**
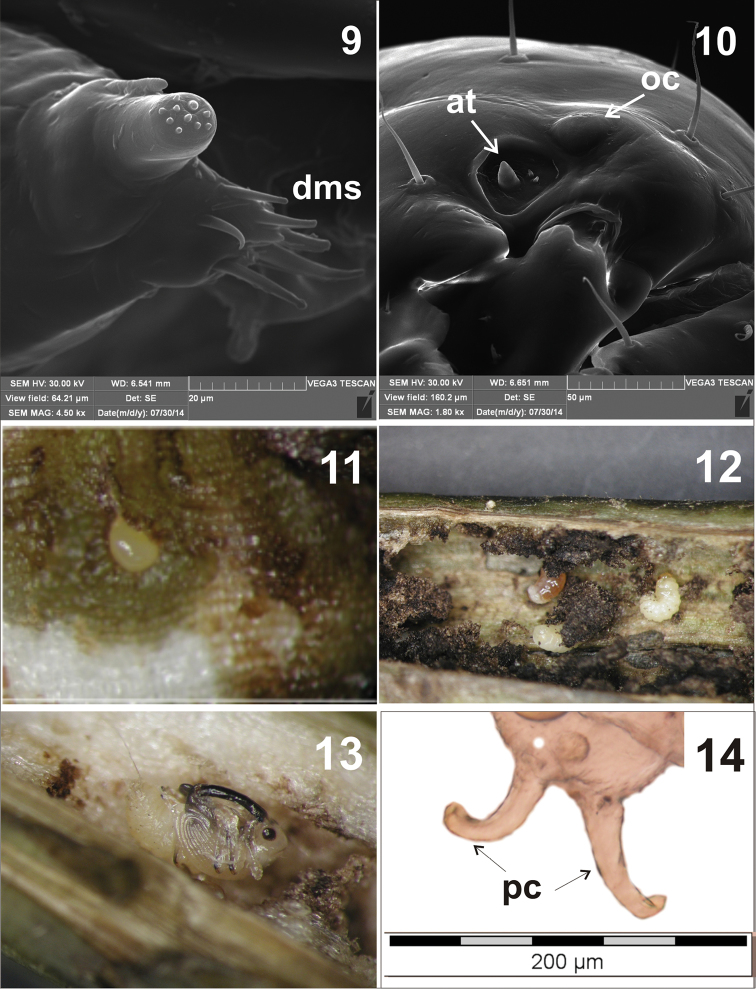
**9** Maxillary palpus (dms – dorsal robust setae) **10** ocellus (oc) and antenna (at) **11** egg **12** L_3_ during construction of the pupal chamber **13** pupa **14** urogomphi (pc).

Body length: ca. 2.67 mm, width ca. 0.94 mm. Colour whitish-grey.

**Head** (ventral view). Rostrum reaching ventrite V, with 1 distinct seta (*rs*) at mid-length, in front of the antennal insertion. Antennae relatively long, club with conical papillae. Antennae sub-parallel to protibia. Frons with 1 pair of setae (*vs*), about as long as rostral setae, situated at the level of the hind margin of the eyes (Figs 6, 7). **Pronotum** length greater than width, with 5 pairs of setae (*as1*, *as2*, *sls*, *pls1*, *pls2*) (Figs 6–8). Setae *as1*and *as2* long and located at the apical margin. Setae *sls* a bit shorter, located at the external margin in the middle of the edge. Setae *pls1and pls2* as long as *sls*, and located close to the back margin. **Mesonotum** short and **metanotum** two times longer than mesonotum. Mesonotum with clearly visible scutellar shield, metanotum with 3 pairs of short setae, medially located. All femora with 1 long, thin seta (*fes1-3*) located apically (Figs 6–8). **Abdomen.** Abdominal tergites I-III with 7 pairs of setae arranged in two rows – 2 closer to the upper edge of the segment, nearly at the external margin, 5 closer to the base. Tergites IV-VI with 5 pairs of setae arranged in two rows – 1 closer to the upper edge of the segment, nearly at the external margin, 4 closer to the base. Tergite VII with 3 pairs of setae arranged in one row, VIII with one pair. Segment IX without setae. Urogomphi (pseudocerci) (*pc*) on abdominal segment IX, laterally parted, crescent-shaped, narrow. Segment X reduced (Figs 6–8, 14). Spiracles on abdominal segments I-VI functional, well visible, positioned longitudinally on pleura (Fig. 6). Gonotheca visibly divided in female, single in male.

### Biological information

According to the available literature, the period of occurrence of adults of *Squamapion elongatum* is April (Cmoluch 1962). During sampling they were found only on meadow sage, although woodland sage was also examined. This confirms Dieckmann’s (1977) observations in Central European conditions that meadow sage is the food and breeding plant of this species. It was observed that fertilized females bit out small holes in the nodes and internodes of the sage stems, in which they laid eggs. Often there were two or more larvae in one place, suggesting that females can lay several eggs in one hole. The oldest larvae were found on the root of the host plant, while the younger larvae were observed higher up, to about 2/3 of the plant height. After an average of 12 days (from 10 to 14 days), the larvae of the first instar (L_1_) hatched and enlarged the egg chamber, making contact with the pith inside the stem. Here the larvae fed intensively, creating tunnels. After about seven days the larvae finished moulting, leading to L_2_. L_2_ fed on average for 10 days, extending the tunnels begun by L_1_. The life span of L_3_ ranged from 10 to 13 days (data based on laboratory cultures and field observations). The greatest weight gain in the larvae occurred during this period, as well as preparation for pupation. The larvae dig a tunnel 6-12 mm in length and use loose fibres to build a pupal chamber (Fig. 12). Initially the pupa was white; later it assumed a creamy shade, and as pigmentation progressed it turned grey. The eyes and rostrum took on the colour first, then the femorotibial articulations and tarsi, followed by the secondary pterothecae, and gradually the rest of the body. The pupal period lasted 12 days on average, and the development cycle of one generation from egg to imago lasted on average 51–54 days.

Larvae of this weevil were attacked by parasitic hymenopterans of the Chalcidoidea. Both internal (*Entedon* Dalman, 1820 sp.) and external (*Trichomalus* Thomson, 1878 sp.) parasites from that superfamily were observed.

## Discussion

This is the first description of the immature stages of a species of the genus *Squamapion*. The features described were compared with those described by Scherf (1964) for the genus *Apion* Herbst, 1797 *sensu lato*, by Marvaldi (2003) for Apioninae, by Łętowski (1991) for *Stenopterapion* Bokor, 1923, *Omphalapion* Schilsky, 1906 and *Hemitrichapion* Voss, 1959, by Gosik et al. (2010) for *Diplapion* Reitter, 1916, by Wang et al. (2013) for *Pseudaspidapion* Wanat, 1990 and by Alonso-Zarazaga and Wanat (2014) for Apioninae Schoenherr, 1823.

The morphology of the egg does not differ substantially from the typical characteristics of the eggs of apionid beetles. The localization of eggs on the plant and the duration of this stage are also similar to those of other apionid beetles with the same lifestyle.

In general the body of larval *Squamapion elongatum* does not deviate from representatives of the subfamily Apioninae described by Alonso-Zarazaga and Wanat (2014). It is very similar to that of larvae of species living inside common sainfoin described by Łętowski (1991) or *Diplapion confluens* (Kirby, 1808) living in the roots of *Anthemis tinctoria* L. (Gosik et al. 2010). The larvae of *Squamapion elongatum* have a typical number of spiracles for Apionidae, as described in Emden (1938), Scherf (1964) and Łętowski (1991), the absence of spiracles on abdominal segment VIII being an important apomorphic character defining the group (as Apioninae in Marvaldi 2003). Larvae of different stages differ from one another in some characteristics, mainly changes in the distribution pattern of the setae. However, no change in the colour of the larvae was observed during growth, unless caused by internal parasitic infection. The differences in the chaetotaxy of the larval body are shown using L_3_ of *Squamapion elongatum*, *Diplapion confluens* and *Pseudaspidapion botanicum* as examples (Table 2).

**Table 2.** Character comparison between *Squamapion elongatum*, *Diplapion confluens* and *Pseudaspidapion botanicum*.

Species		*Squamapion elongatum*	*Diplapion confluens*	*Pseudaspidapion botanicum*
arrangement of setae *pes* on epicranium		4 pairs of minute posterior setae (*pes*1–4) separated from one pair of longer setae (*pes*5) and 1 pair of sensilla	2 pairs (*pes*1, *pes*2) short and blunt	6 pairs of setae, *pes*1 shortest, *pes*2–6 successively longer
number of setae on maxillary palpus	basal segment	3 very short setae	1 long and 1 micro seta	1 short inner seta and 1 sensillum
	distal segment	1 short sensilla	none	1 crenulate seta
length of labral rods (*lmr*)		rather short	long	long
occurrence *pms*2		present	present	absent
number of setae on the mandibles		2	1	1
number of conical papillae	*dms*	8	5	5
	*vms*	0	2	4
number of setae (*pns*) on pronotum (L_3_)		5	4	6

The characteristics of the pupae of this weevil species do not differ from other representatives of Apionidae, except for the presence of a mesofemoral seta, also found in *Diplapion* but not in *Pseudaspidapion*, and the lack of a pair of setae on the 8th abdominal tergite, in contrast to one pair of setae in *Pseudaspidapion* and two in *Diplapion*. Relatively long urogomphi flared to the sides were present, as in *Pseudaspidapion* and *Diplapion* (Gosik et al. 2010; Wang et al. 2013).

The laboratory work confirmed that the food and breeding plant for this species is meadow sage (*Salvia pratensis*). The preimaginal development of *Salvia elongatum* occurs in the nodes and internodes of the stems, as well as in the basal part of the root.
